# A Fast and Robust Ellipse-Detection Method Based on Sorted Merging

**DOI:** 10.1155/2014/481312

**Published:** 2014-03-23

**Authors:** Gangyi Wang, Guanghui Ren, Zhilu Wu, Yaqin Zhao, Lihui Jiang

**Affiliations:** School of Electronics and Information Engineering, Harbin Institute of Technology, Harbin, Heilongjiang 150001, China

## Abstract

A fast and robust ellipse-detection method based on sorted merging is proposed in this paper. This method first represents the edge bitmap approximately with a set of line segments and then gradually merges the line segments into elliptical arcs and ellipses. To achieve high accuracy, a sorted merging strategy is proposed: the merging degrees of line segments/elliptical arcs are estimated, and line segments/elliptical arcs are merged in descending order of the merging degrees, which significantly improves the merging accuracy. During the merging process, multiple properties of ellipses are utilized to filter line segment/elliptical arc pairs, making the method very efficient. In addition, an ellipse-fitting method is proposed that restricts the maximum ratio of the semimajor axis and the semiminor axis, further improving the merging accuracy. Experimental results indicate that the proposed method is robust to outliers, noise, and partial occlusion and is fast enough for real-time applications.

## 1. Introduction

Shape-based object detection is an important topic in the field of computer vision. Because they are a commonly appearing shape, ellipses are often seen in many types of artificial and real scenes. Therefore, ellipse detection has many applications, including detecting industrial components, iris detection, and detecting traffic signs. Moreover, ellipse detection is often used as a component of other computer vision tasks, such as 3D calibration [1] and object recognition [2]. However, it is a challenging task to detect ellipses in complex scenes. The major difficulties are as follows.

**Figure 1:**
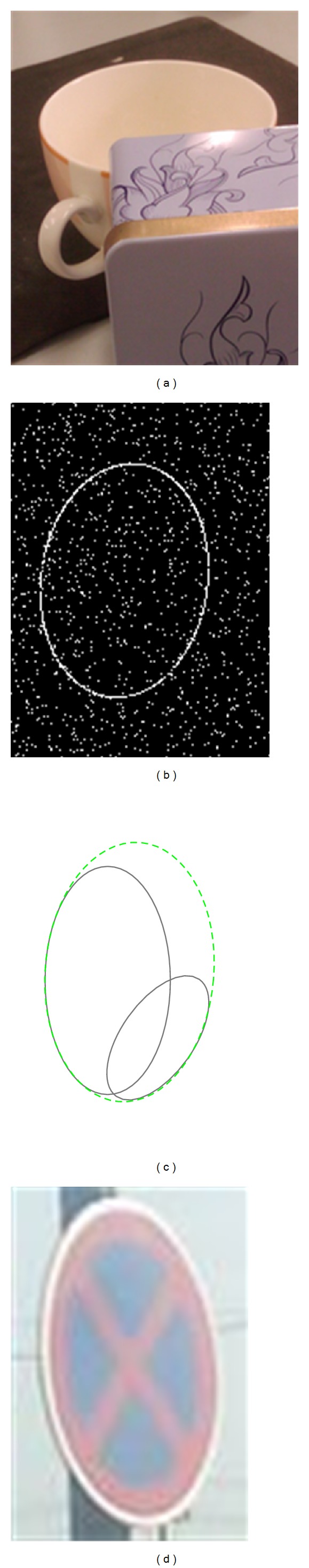
Major difficulties of ellipse detection. (a) Ellipse at the cup mouth is blocked partially. (b) Noise interrupts the elliptical curve or generates fake branches. (c) Outlier causes incorrect detection, where the black ellipses are true ones and the green dashed ellipse is the incorrect detection result. (d) Low resolution makes the ellipse curves imprecise.

1. In real scenes, elliptical objects are often blocked by other objects, which renders the ellipses incomplete, as shown in Figure 1(a). It is difficult to accurately extract the ellipses if they are blocked significantly.
2. Noise is a common problem in image processing. A large amount of noise can blur or destroy an elliptical curve and can increase the false negative ratio, as shown in Figure 1(b).
3. There are often curves from other objects that are close to ellipses in complex scenes, which we refer to as outliers. Sometimes the outliers form approximate elliptical curves together with partial curves of true ellipses, which can increase both the false negative and false positive ratio, as shown in Figure 1(c).
4. For digital images, the position of an edge pixel often differs slightly from its real position, which is caused by quantization error. This phenomenon is especially evident in low-quality images, as shown in Figure 1(d).
5. For real-time applications, the performance of the detection method should be at least several or even dozens of frames per second. How to increase the detection speed while keeping the accuracy acceptable is another difficulty.

There are three basic classes of ellipse-detection approaches: Hough transform (HT) based methods, genetic algorithm (GA) based methods, and edge-following methods.

The Hough transform [3] is a widely used method for detecting parameterized curves such as lines and circles. This method first votes on the parameter space with edge pixels and then obtains candidates by searching for the parameters with high votes. The advantage of HT-based methods is their robustness to noise and partial occlusion. However, the 5D parameter space needed for ellipse detection consumes a very large amount of memory and computation, which makes the standard Hough transform unusable in most cases.

Many improved methods have been proposed to reduce the dimension of the parameter space. Some of the methods [4–8] first vote for a portion of the parameters (such as the center coordinates) by utilizing properties of ellipses and then search for other parameters. However, the accuracy of these methods tends to deteriorate when the required properties are not met, under some adverse conditions (such as partial occlusion) [9]. The method in [10] votes on edge pixels that are selected with the principle of convexity, distance, and intersection angle. However, this method relies on the gradient directions of the edge pixels, which are not always available. In [11, 12], the randomized Hough transform is proposed, which votes for ellipses with three randomly selected edge pixels and continually updates the parameters of the detected ellipses. This method runs fast and has a low requirement for memory. However, it is sensitive to noise and outliers [13].

Another class of ellipse detection methods is based on the genetic algorithm [14–16]. The genetic algorithm (GA) is a method for solving optimization problems. When used in ellipse detection, the ellipse parameters are usually coded as chromosomes, and the best parameters are gradually evolved by iteratively exchanging chromosomes within the population. GA-based methods are usually robust to partial occlusion, but only one ellipse can be detected at a time. Therefore, the optimization process must be performed multiple times when detecting more than one ellipse, which makes the method time consuming. In addition, nearby outliers often cause the method to become stuck in a local optimum, which reduces the detection accuracy.

Edge following is the third class of ellipse detection methods. The basic idea of these methods is to gradually group edge pixels into line segments, elliptical arcs, and, finally, ellipses. An early representative method of this class is the Upwrite method [17], which groups edge pixels with similar local properties and classifies each group as a line, circle, or ellipse. This method is fast and can detect multiple classes of curves concurrently, but the accuracy deteriorates substantially when the noise and outliers increase. The method in [18] links edge pixels to arcs, calculates the fitness of each pair of arcs, and then continually merges the pairs that have fitness values greater than a threshold until no more pairs can be merged. This method is robust to partial occlusion but is slow and sensitive to outliers. The methods in [19, 20] first link edge pixels to line segments, then merge line segments into elliptical arcs, and finally merge the arcs into ellipses. During the merging process, both of the methods filter out line segment pairs that do not satisfy some of the constraints; thus, the detection speed is increased. However, the line segments/elliptical arcs are sometimes incorrectly merged because the merging order is arbitrarily selected. The method in [9] is the most complex edge-following method at present. It adopts an optimization algorithm during the merging process and iteratively corrects the detection result. This method has high detection accuracy and is robust to many adverse situations, but the computational complexity is too high for real-time applications.

There are also hybrid methods that combine several methods from the three classes. The method in [21] detects circular arcs with Hough transform and grows the arcs along edge pixels; then, it fits the grown arcs to ellipses. Although the memory consumption of the method is not very large, the computational complexity is still high because of the iterative process of edge growing and fitting. The method in [22] combines an edge-following method with Hough transform; this method is robust to partial occlusion and outliers, but it runs slowly.

In summary, most of the ellipse-detection methods have limitations; they either are not robust enough in complex scenes or are too slow for real-time applications and embedded systems. In this paper, we propose a fast and robust ellipse-detection method that is based on sorted merging and can be classified as an edge-following approach. Compared to existing edge-following methods, the proposed method introduces a sorted merging strategy that utilizes more global information and significantly increases the merging accuracy. Moreover, many types of filtering strategies are used, which effectively speed up the merging process. In addition, an ellipse-fitting method with a limited axial ratio is proposed, which further increases the detection accuracy because the ellipses to be detected are usually not very flat in most of the applications.

The remainder of this paper is organized as follows. In Section 2, the core concept and the details of the proposed method are described. In Section 3, the detection accuracy and the efficiency of the proposed method are evaluated and compared with other existing methods. In Section 4, conclusions are drawn and possible improvements for the future are presented.

## 2. The Ellipse-Detection Method Based on Sorted Merging

**Figure 2:**
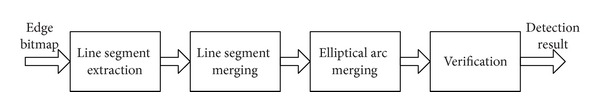
Block diagram of the proposed method.

The proposed method comprises four steps, as shown in Figure 2. The input for the method is the edge bitmap of an image. The edge pixels are first linked into strips; then, the strips are split into short line segments, which are merged into elliptical arcs in the next step. Elliptical arcs are further merged into candidate ellipses, and finally, the candidate ellipses are verified in the edge bitmap. Ellipses that pass verification are output as detection results.

The method uses a bottom-up technique, whereby edge pixels without any structural information are gradually merged into lines, arcs, and ellipses. Each step the method moves upward; the complexity of a single object (pixel, line, arc, and ellipse) increases while the number of objects decreases. As a result, the computational complexity of each step is retained at a moderate level, which makes the method efficient. Each step is described in detail in the following sections.

### 2.1. Line Segment Extraction

Line segment extraction is the first step of the method. This step aims at representing the edge bitmap with a series of line segments while preserving most of the information from the edge bitmap. The detailed process includes the following steps.

**Figure 3:**
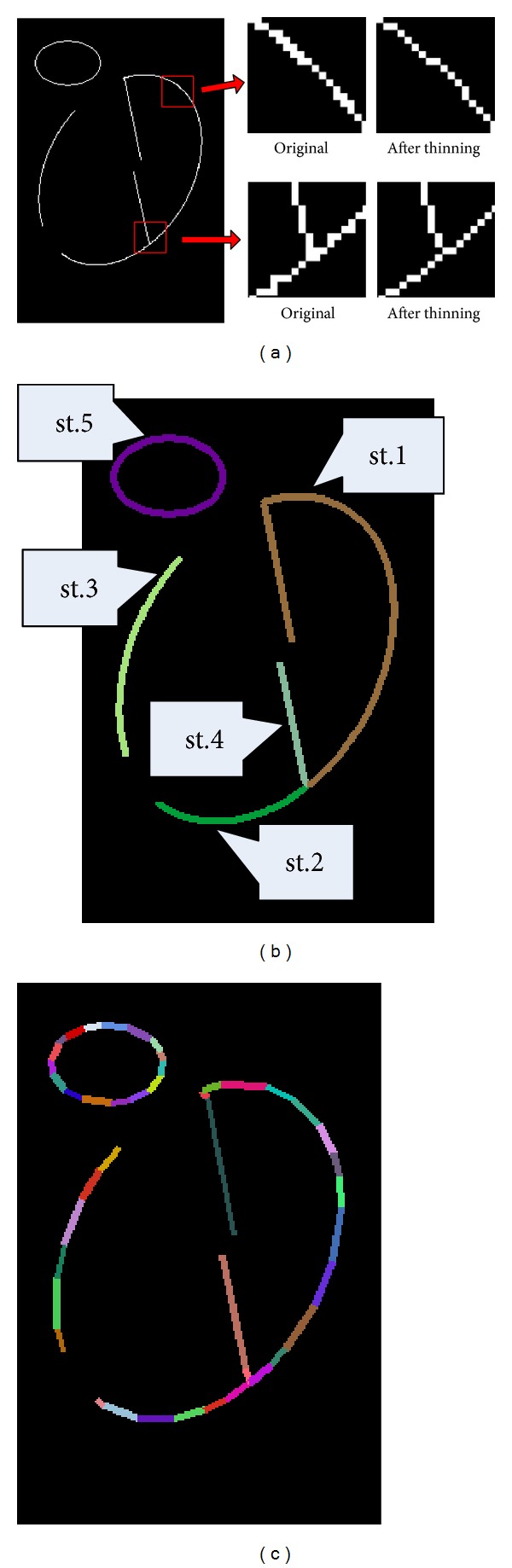
The process of line segment extraction. (a) Number of neighboring pixels decreases after morphological thinning. (b) Extracted strips after edge linking; each color corresponds to a strip. (c) Line segments extracted from strips; each color corresponds to a line segment.

1. Edge bitmap thinning: there are usually redundant connections in edge bitmaps; these connections can be removed by a morphological thinning operation. With this step, the number of neighboring pixels owned by each pixel can be reduced to a minimum, as shown in Figure 3(a).
2. Link the edge pixels into strips with no branches: the number of neighboring pixels owned by an edge pixel should be within one of the following cases. Most of the edge pixels have two neighbors, which make up the intermediate points of the strips; pixels with one or more than two neighbors are the end points of the strips; and pixels with no neighbors are isolated pixels that can be removed directly. The linking process starts from one end point and generates strips along the direction of each neighboring pixel; each strip keeps growing until it meets an end point. There can also be loops without end points. Therefore, an extra step to extract all of the loops is executed; this step arbitrarily selects an intermediate point that is not linked as the starting point of a strip and grows the strip along edge pixels until it meets the starting point again. All of the strips extracted from Figure 3(a) are shown in Figure 3(b).
3. Split the strips into line segments: in an ideal situation, ellipses can be directly extracted to determine whether each strip is an ellipse or not. However, with the interference of outliers, elliptical arcs are often linked with curves from other objects, which leads to an incorrect determination. Strip 1 in Figure 3(b) is an example of this situation. To break up the connections of ellipses and outliers, we split the strips into line segments. The method in [23] is employed to complete this step. The set {*l*_*i*_} of the extracted line segments is shown in Figure 3(c).

### 2.2. Line Segment Merging

The number of line segments can be up to several thousand or more when the edge bitmap is complex. Therefore, it is very time consuming to directly determine whether each pair of line segments can be merged or not. To improve the efficiency, the merging process is divided into two steps: line segment merging and elliptical arc merging. The former step is more local and merges only nearby objects, while the latter step processes objects more globally. In this way, the number of objects to be processed in each step is decreased.

However, dividing the merging process into two steps causes errors from the former step to be directly introduced into the latter step and leads to incorrect detection results in the end. Therefore, the ratio of incorrect merging should be as low as possible in the former step. We use a sorted merging strategy to take more global information into account. To facilitate the description of the method, we make the following definition of a line and a pair of lines.

**Definition 1**

**Figure 4:**
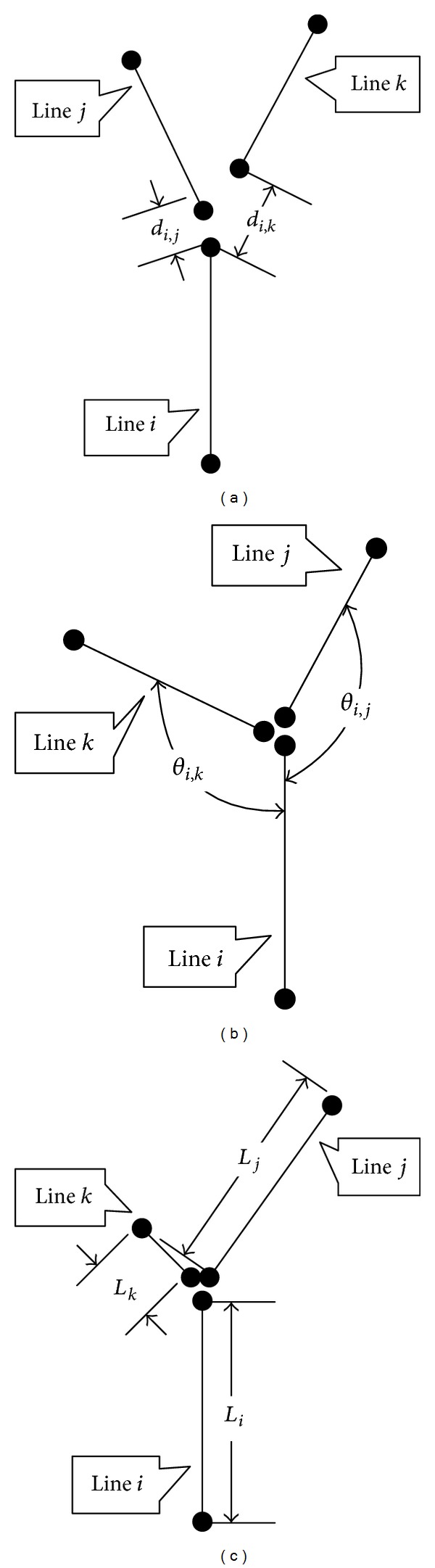
Relationship and merging degree between line segments. (a) Distance between line segments *i* and *j* is shorter, corresponding to higher merging degree. (b) Intersection angle between *i* and *j* is larger, corresponding to higher merging degree. (c) Lengths of line segments *i* and *j* are more similar, corresponding to higher merging degree.

The distance *d*_*i*,*j*_ of the two line segments *l*_*i*_ and *l*_*j*_ is

(1) $$\begin{array}{c} d_{i,j}=\underset{m,n}{\min}\left(\sqrt{{\left(x_{i}^{m}-x_{j}^{n}\right)}^{2}+{\left(y_{i}^{m}-y_{j}^{n}\right)}^{2}}\right), \end{array}$$

where *m*, *n* ∈ {1,2} denotes one of the two end points of a line segment and *x*_*i*_^*m*^, *y*_*i*_^*m*^ denote the horizontal and vertical ordinates of end point *m* of line segment *l*_*i*_, as shown in Figure 4(a).

**Definition 2**

The intersection angle *θ*_*i*,*j*_ of the line segments *l*_*i*_ and *l*_*j*_ is the angle between the two vectors that correspond to *l*_*i*_ and *l*_*j*_, and the starting points of the two vectors are the pair of end points that have the shortest distance, as shown in Figure 4(b).

**Definition 3**

The length of line segment *l*_*i*_ is the Euclidean distance of the two end points of *l*_*i*_, as shown in Figure 4(c).

The entire line segment merging process comprises the following steps.

(1) Form a set {*LP*_*i*,*j*_} of line segment pairs by selecting all of the pairs (*l*_*i*_, *l*_*j*_) that satisfy the two conditions: *d*_*i*,*j*_ < *d*_max⁡_ and *θ*_*i*,*j*_ > *θ*_min⁡_, where *d*_max⁡_ and *θ*_min⁡_ are the maximum distance and the minimum intersection angle of the two line segments, respectively.

(2) Calculate the merging degree *D*_*i*,*j*_ of each (*l*_*i*_, *l*_*j*_) in {*LP*_*i*,*j*_} with the following equation:

(2) $$\begin{array}{c} D_{i,j}=\frac{1}{d_{i,j}^{2}+1}\cdot \cos\left(\pi -\theta_{i,j}\right)\cdot \frac{m_{i,j}}{M_{i,j}}, \end{array}$$

 where *m*_*i*,*j*_ = min⁡(*L*_*i*_, *L*_*j*_) and *M*_*i*,*j*_ = max⁡(*L*_*i*_, *L*_*j*_). With the equation above, the pair of line segments with smaller *d*_*i*,*j*_, larger *θ*_*i*,*j*_, and more similar lengths has a larger merging degree, as shown in Figures 4(a)–4(c).

(3) Split {*LP*_*i*,*j*_} into two sets {*LP*_*i*,*j*_^(1)^} and {*LP*_*i*,*j*_^(2)^} according to *m*_*i*,*j*_ of the pairs. Any pair that satisfies *m*_*i*,*j*_ ≥ *Th*_short_ is included in {*LP*_*i*,*j*_^(1)^}; otherwise, it is included in {*LP*_*i*,*j*_^(2)^}. It can be proved that the length of a chord for an ideal ellipse satisfies the following condition:

(3) $$\begin{array}{c} L\geq 2\beta \sqrt{1-{\left(d-\frac{1}{\alpha }\right)}^{2}}, \end{array}$$

where *α* and *β* denote the semimajor axis and semiminor axis of the ellipse, respectively, and *d* is the maximum distance between the chord and the elliptical arc that corresponds to the chord. According to formula (3), the line segments that lie on an ellipse should not be too short. Therefore, line segment pairs in {*LP*_*i*,*j*_^(2)^} with a smaller *m*_*i*,*j*_ than those in {*LP*_*i*,*j*_^(1)^} are unlikely to be merged. Either they are not lying on ellipses or other objects are interfering with them. We process pairs in {*LP*_*i*,*j*_^(2)^} after no more pairs in {*LP*_*i*,*j*_^(1)^} can be merged, to prevent the pairs with higher merging probability from being interfered by pairs with lower merging probability.

**Figure 5:**
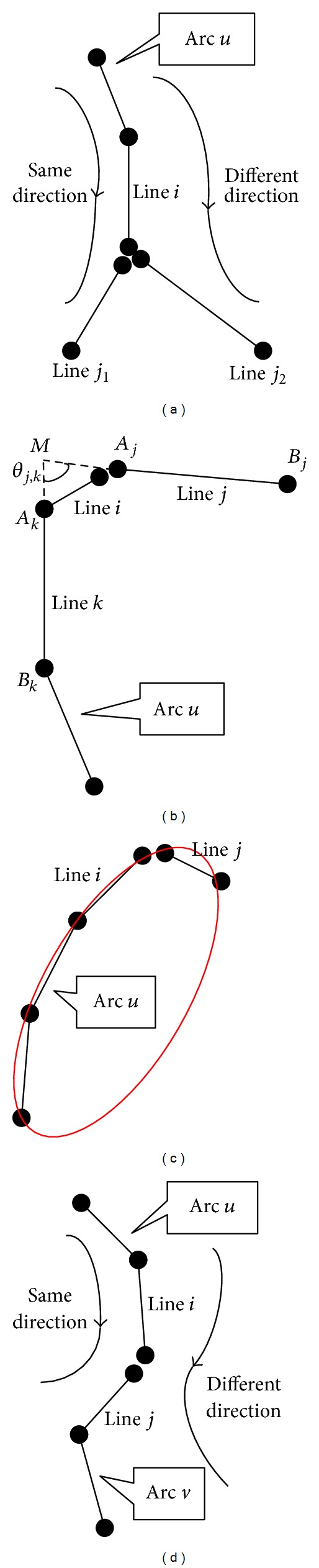
Illustrations of line segment merging conditions, where linked line segments denote elliptical arcs. (a) Rotating direction condition; line *j*_1_ and arc *u* are of the same rotating direction, satisfying the condition, while line *j*_2_ and arc *u* do not. (b) The length and angle condition; line *j* and line *j* generate a shape angle, not satisfying the condition. (c) The fitting condition; the fitting score of line *j* is too low, not satisfying the condition. (d) The rotating direction condition for arcs; line *j* and arc *u* are of the same rotating direction, but line *i* and arc *v* are not, preventing the two arcs from merging.

(4) Take out the pairs in {*LP*_*i*,*j*_^(1)^} sequentially according to the descending order of *D*_*i*,*j*_. The two line segments *l*_*i*_ and *l*_*j*_ of pair (*l*_*i*_, *l*_*j*_) are merged if the specific conditions described later are met. During the merging process, there are three situations, as follows.

(i) Merging of two line segments: if both line segments *l*_*i*_ and *l*_*j*_ are not merged with other line segments, then directly create a new elliptical arc *a*_*u*_ with line segments *l*_*i*_ and *l*_*j*_.
(ii) Merging of a line segment and an elliptical arc: if one of the line segments (suppose it is *l*_*i*_) has joined an elliptical arc *a*_*u*_, while the other segment (suppose it is *l*_*j*_) has not, then merge *l*_*j*_ into *a*_*u*_ if the following three conditions are met.
  (a) The condition of rotating direction: the rotating direction from *l*_*j*_ to *l*_*i*_ should be the same as the rotating direction from *l*_*i*_ to other line segments in *a*_*u*_, as shown in Figure 5(a).
  (b) The condition of length and angle: line segment *l*_*j*_ and any line segment *l*_*k*_ in *a*_*u*_ should satisfy the following condition:
    (4) $$\begin{array}{c} \frac{\left|A_{j}B_{j}\right|+\left|A_{k}B_{k}\right|}{\left|MB_{j}\right|+\left|MB_{k}\right|}<0.7\;\text{or}\;\theta_{j,k}>90^{{}^{\circ}}, \end{array}$$
   where *A*_*j*_, *B*_*j*_, *A*_*k*_ and *B*_*k*_ are the end points of *l*_*j*_ and *l*_*k*_ and *M* is the intersection of *l*_*j*_ and *l*_*k*_, as shown in Figure 5(b). With this condition, it is ensured that two line segments forming a shape angle will not be merged into the same elliptical arc.
  (c) The fitting condition: fit an ellipse *E* with all of the edge pixels contained in *a*_*u*_ and *l*_*j*_ and calculate the fitting score of each edge pixel *x* with the following formula:
    (5) $$\begin{array}{c} s\left(x\right)=\{\begin{array}{cc} 1, & \min\left({||x-y||}_{2}\right)\leq 1\;\forall y\in E\\ 0, & \text{otherwise}. \end{array} \end{array}$$
 Next, obtain the fitting scores $\bar{S_{u}}$ and $\bar{S_{j}}$ of *a*_*u*_ and *l*_*j*_ by calculating the mean fitting scores of all of the edge pixels contained in *a*_*u*_ and *l*_*j*_. If $\bar{S_{u}}\geq Th_{\text{fit}}$ and $\bar{S_{j}}\geq Th_{\text{fit}}$, then the fitting condition is met, where *Th*_fit_ is a threshold that can be set to 0.8 or 0.9. With this condition, it is ensured that most of the edge pixels in *a*_*u*_ and *l*_*j*_ are close to the fitted ellipse, as shown in Figure 5(c). Details about the fitting method are described in Section 2.5.
(iii) Merging of two elliptical arcs: if line segments *l*_*i*_ and *l*_*j*_ have joined elliptical arcs *a*_*u*_ and *a*_*v*_, respectively, then merge *a*_*v*_ into *a*_*u*_ if the following three conditions are met.
  (a) The rotating condition: the rotating direction from *l*_*j*_ to *l*_*i*_ should be the same as the rotating direction from *l*_*i*_ to other line segments in *a*_*u*_; at the same time, the rotating direction from *l*_*i*_ to *l*_*j*_ should be the same as the rotating direction from *l*_*j*_ to the other line segments in *a*_*v*_.
  (b) The condition of length and angle: similar to condition (ii) for the merging of a line segment and an elliptical arc, this condition requires that any two line segments from *a*_*u*_ and *a*_*v*_, respectively, should not be forming a sharp angle.
  (c) The fitting condition: similar to condition (iii) for the merging of a line segment and an elliptical arc, this condition requires that both fitting scores of $\bar{S_{u}}$ and $\bar{S_{v}}$ are smaller than *Th*_fit_.

(5) After all of the line segment pairs in {*LP*_*i*,*j*_^(1)^} have been processed, the pairs in {*LP*_*i*,*j*_^(2)^} begin to be processed using the same criteria as in step (4).

(6) When all of the line segment pairs have been processed, a series of elliptical arcs are generated. We select the arcs that contain at least *LC*_min⁡_ line segments and obtain a set {*a*_*i*_} of elliptical arcs; then, we output {*a*_*i*_} for the next stage.

**Figure 6:**
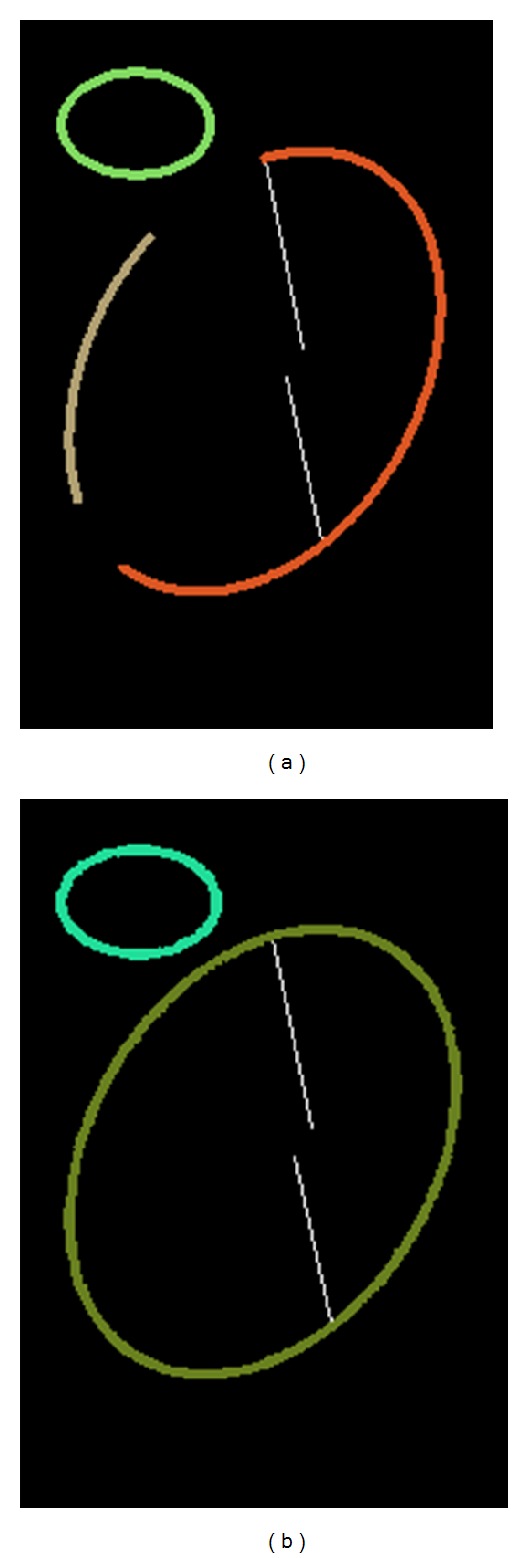
Results of line segment merging and elliptical arc merging. (a) Result of line segment merging. (b) Result of elliptical arc merging.

Figure 6(a) shows all of the elliptical arcs that are generated after line segment merging. By calculating the merging degree of the line segments, the line segments with a higher merging probability are merged first, which effectively prevents the line segments from being incorrectly merged in an arbitrary order.

### 2.3. Elliptical Arc Merging

The line segment merging step generates elliptical arcs with nearby line segments. However, an ellipse can be segmented into several arcs by noise or partial occlusion, as shown in Figure 6(a). Therefore, an extra step is needed, which merges all of the elliptical arcs that belong to the same ellipse. We make the following definition to facilitate the description of the method.

**Definition 4**

Suppose that all of the line segments contained in the elliptical arc *a*_*i*_ are sequentially *l*_*i*,1_, *l*_*i*,2_, ..., *l*_*i*,*n*_, and the intersection angles between the line segments and the *x*-axis are *θ*_*i*,1_, *θ*_*i*,2_, ..., *θ*_*i*,*n*_, respectively; then, the rotating degree of *a*_*i*_ is defined as deg⁡(*a*_*i*_) = ∑_*k*=1_^*n*−1^|*θ*_*i*,*k*+1_ − *θ*_*i*,*k*_|, and the rotating range of *a*_*i*_ is defined as ⋃_*k*=1_^*n*−1^[*θ*_*i*,*k*_, *θ*_*i*,*k*+1_].

Similar to the concept of line segment merging, elliptical arcs are also merged in the descending order of merging degrees. The detailed steps are as follows.

**Figure 7:**
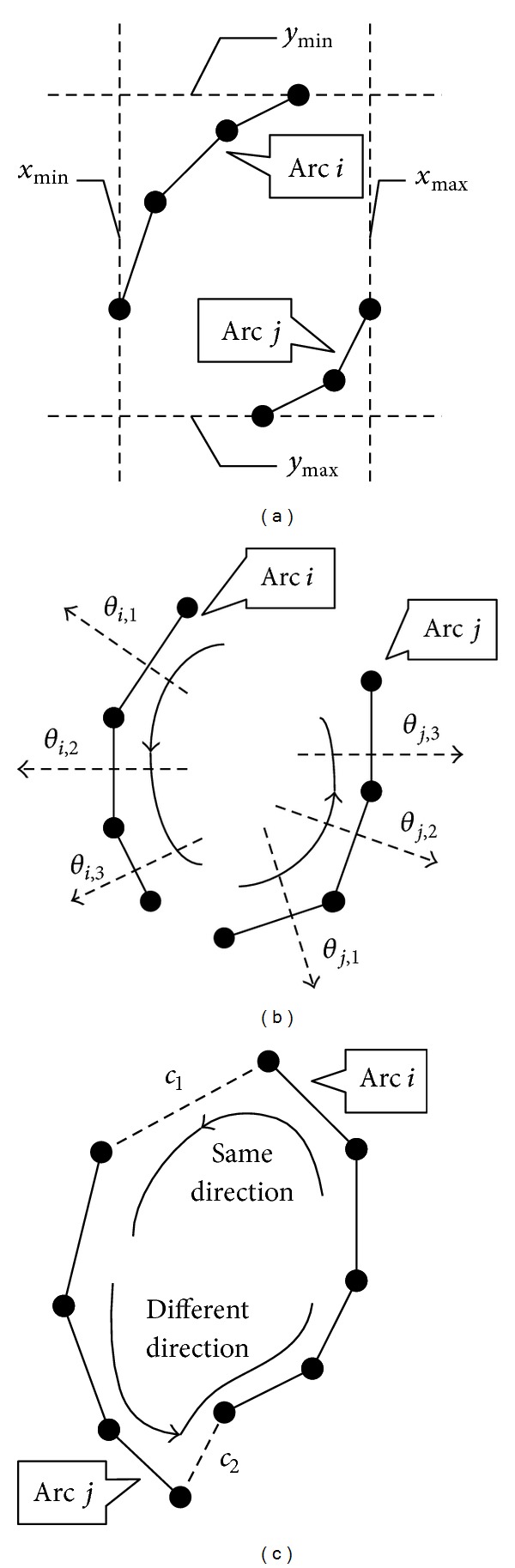
Illustrations of elliptical arc merging conditions. (a) The condition of distance. (b) The condition of coinciding degree, which is 0 for arc *i* and arc *j*. (c) The condition of rotating direction; chord *c*_2_ makes the rotating direction of the whole curve not consistent.

(1) Form a set {*AP*_*i*,*j*_} of elliptical arc pairs by selecting all of the pairs (*a*_*i*_, *a*_*j*_) that satisfy all of the following four conditions.
  (a) The condition of distance: in real-life applications, the size of the ellipses to be detected is usually limited. For a detection task with a maximum semimajor axis *α*_max⁡_, two arcs *a*_*i*_ and *a*_*j*_ should satisfy the following distance condition:
    (6) $$\begin{array}{c} x_{\max}-x_{\min}\leq 2\alpha_{\max}\\ y_{\max}-y_{\min}\leq 2\alpha_{\max}, \end{array}$$
   where *x*_max⁡_, *x*_min⁡_, *y*_max⁡_, and *y*_min⁡_ are the maximum and minimum of the horizontal and vertical ordinates of all of the edge pixels that are contained in *a*_*i*_ and *a*_*j*_, as shown in Figure 7(a).
  (b) The condition of coinciding degree: there should be no intersection between the rotating ranges of *a*_*i*_ and *a*_*j*_, which belong to the same ellipse, as shown in Figure 7(b). To be robust to noise, we set a threshold *Th*_DI_ to be the maximum coinciding degree, where *Th*_DI_ = min⁡(deg⁡(*a*_*i*_), deg⁡(*a*_*j*_))/10.
  (c) The condition of rotating direction: the two arcs can be connected with two chords *c*_1_ and *c*_2_, to be transformed into a closed curve, as shown in Figure 7(c). The rotating direction throughout the curve should be consistent if *a*_*i*_ and *a*_*j*_ belong to the same ellipse.
  (d) The fitting condition: similar to that of the line segment merging, the fitting condition requires both of the fitting scores *a*_*i*_ and *a*_*j*_ to be not smaller than *Th*_fit_.
(2) Calculate the merging degree *F*_*i*,*j*_ of each pair (*a*_*i*_, *a*_*j*_) in {*AP*_*i*,*j*_} with the following formula:
  (7) Fi,j=min⁡(Si¯,Sj¯)·min⁡[len(ai),len(aj)]peri(Ei,j) ·[deg⁡(ai)+deg⁡(aj)],
 where len(*a*_*i*_) is the length of *a*_*i*_ and peri(*E*_*i*,*j*_) is the perimeter of ellipse *E*_*i*,*j*_ fitted with *a*_*i*_ and *a*_*j*_.
(3) Initialize a candidate ellipse set {*ce*_*i*_}; each member in {*ce*_*i*_} corresponds to a unique elliptical arc in the set {*a*_*i*_}.
(4) Take out the pairs in {*AP*_*i*,*j*_} sequentially in the descending order of *F*_*i*,*j*_. Suppose that the candidate ellipses *ce*_*i*_ and *ce*_*j*_ in {*ce*_*i*_} correspond to a pair of elliptical arcs *a*_*i*_ and *a*_*j*_, respectively; *ce*_*j*_ are merged into *ce*_*i*_ if the following two conditions are met.
  (a) The condition of conflicting arc pairs: assuming that all of the arcs contained in *ce*_*i*_ and *ce*_*j*_ form two sets {*a*_*u*_} and {*a*_*v*_}, respectively, then any arc pair (*a*_*u*_, *a*_*v*_) should be a member of {*AP*_*i*,*j*_}, where *a*_*u*_ ∈ {*a*_*u*_} and *a*_*v*_ ∈ {*a*_*v*_}. Because any pair of elliptical arcs that might be from the same ellipse is included in {*AP*_*i*,*j*_}, this condition ensures that no conflicting arcs can be merged into the same ellipse.
  (b) The fitting condition: the fitting score $\bar{S_{k}}$ of any *a*_*k*_ ∈ {*a*_*u*_} ∪ {*a*_*v*_} should not be smaller than *Th*_fit_.

After finishing the merging of the elliptical arcs, we obtain a new candidate ellipse set {*e*_*i*_}, as shown in Figure 6(b). Because the four conditions in step (1) are sorted in the ascending order of computational complexity, the pairs of elliptical arcs that cannot be merged are filtered out with a high efficiency, while merging in the descending order of *F*_*i*,*j*_ ensures high merging accuracy.

### 2.4. Ellipse Verification

Candidate ellipses in set {*e*_*i*_} need an extra verification step before they are determined to be true ellipses. The process is as follows.

1. Fit an ellipse *E*_*i*_ with all of the edge pixels that are owned by the candidate ellipse *e*_*i*_.
2. If the semimajor axis does not satisfy *α*_min⁡_ ≤ *α* ≤ *α*_max⁡_, then drop *e*_*i*_, where *α*_min⁡_ and *α*_max⁡_ are the maximum and minimum of the semimajor axis, respectively.
3. If the semimajor axis *α* and semiminor axis *β* of *E*_*i*_ do not satisfy *α*/*β* ≤ *r*_max⁡_, then drop *e*_*i*_, where *r*_max⁡_ is the maximum ratio of the semimajor axis and the semiminor axis.
4. If the length of *e*_*i*_ does not satisfy len(*e*_*i*_)/peri(*E*_*i*_) > *Th*_len_, then drop it; otherwise, join *E*_*i*_ into the ellipse set {*E*_*i*_}.

After the verification step, all of the ellipses in {*E*_*i*_} are the final detection result.

### 2.5. The Ellipse-Fitting Method

Throughout the line segment merging and elliptical arc merging steps, the ellipse-fitting method is frequently used. This method fits a set of edge pixels into an ellipse. The least squares method is a commonly used approach for ellipse fitting. The basic idea is to represent an ellipse with the following equation [24]:

(8) $$\begin{array}{c} F\left(x,y\right)=ax^{2}+bxy+cy^{2}+dx+ey+f=0, \end{array}$$

where *x*, *y* are the horizontal and vertical ordinates of a point and *a*,  *b*,  *c*,  *d*,  *e*,  *f* are ellipse parameters. Let vector **a** = [*a* *b* *c* *d* *e* *f*]^*T*^; for a set of points (*x*_*i*_, *y*_*i*_), the parameter vector a* of the best fitting ellipse can be found by minimizing the following formula:

(9) $$\begin{array}{c} a^{*}=\arg\underset{a}{\min}\underset{i}{\sum }F{\left(x_{i},y_{i}\right)}^{2}. \end{array}$$

The solution can be found by solving a set of linear equations without any iterations, which makes the least squares method very efficient. However, vector a* found by the method might also correspond to a hyperbola or a parabola, which means a failed fitting. To ensure that the fitting result is an ellipse, the method proposed in [24] adds a constraint, 4*ac* − *b* ^2^ = 1, which is based on formula (9), the solution of which can be found by solving a generalized eigenfunction. The numerical stability of the method is improved in [25] by reducing the order of the eigenfunction. Because the method is ellipse-specific and is also efficient, it has been employed in many applications [9, 26].

**Figure 8:**
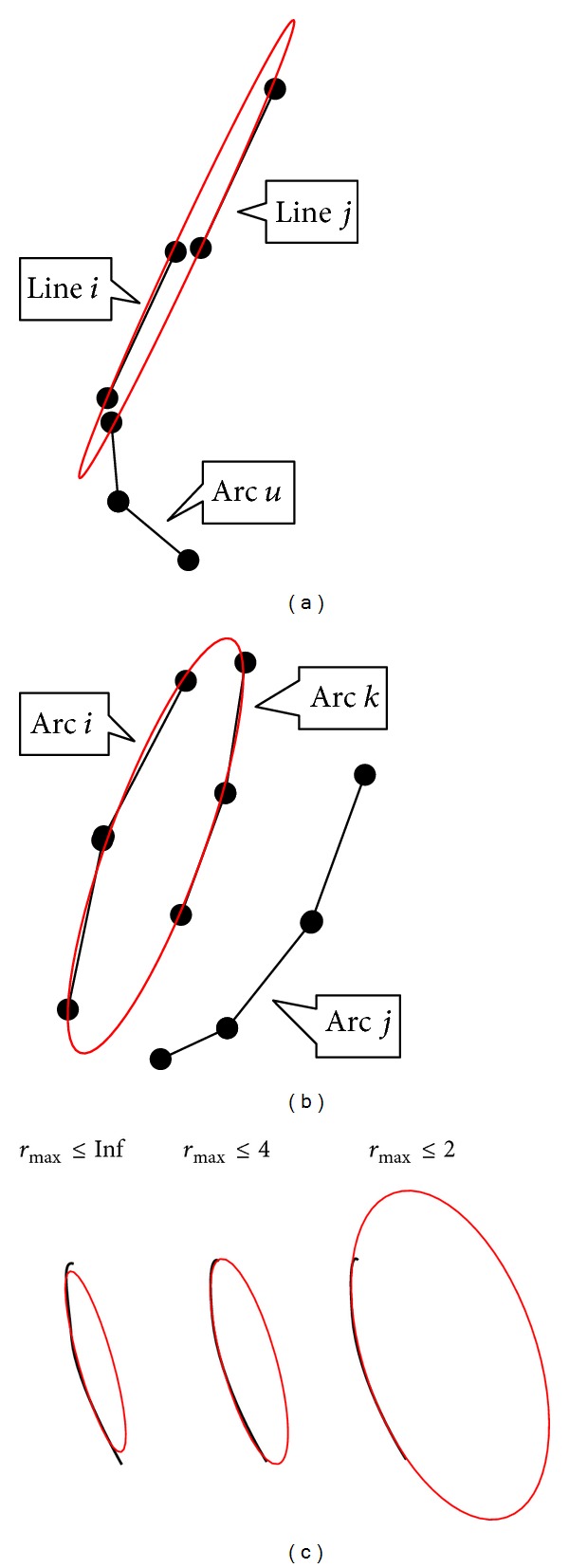
Problems of regular ellipse fitting methods and the fitting results with different *r*_max⁡_. (a) Line *i* should be merged with Arc *u* but is incorrectly merged with Line *j* into a flat ellipse. (b) Arc *i* should be merged with Arc *j* but is incorrectly merged with Arc *k*. (c) Ellipse fitting results with *r*_max⁡_ = +*∞*, *r*_max⁡_ = 4, and *r*_max⁡_ = 2 by the proposed fitting method.

However, the method in [24] sometimes obtains an undesired result when it is applied in the merging process of edge-following ellipse-detection methods. Line segments that should not be merged often fit into a flat ellipse perfectly, as shown in Figure 8(a). The same phenomenon also occurs for low-curvature elliptical arcs, as shown in Figure 8(b). Such cases lower the detection accuracy directly. Because the ellipses to be detected in real-life applications are usually not very flat, we propose an ellipse-fitting method that restricts the maximum *α*/*β* of the fitted ellipse, so that the cases in Figures 8(a) and 8(b) will not occur. The goal of the method can be expressed by solving the following problem:

(10) argmin⁡a ∑iF(xi,yi)2subject  to {4ac−b2=1αβ≤rmax⁡,

where *α* and *β* are the semimajor axis and semiminor axis, respectively.

According to the Kuhn-Tucker conditions, the solution of formula (10) must be one of the solutions of the two following problems:

(11) argmin⁡a ∑iF(xi,yi)2subject  to 4ac−b2=1,

(12) argmin⁡a ∑iF(xi,yi)2subject  to {4ac−b2=1αβ=rmax⁡.

Formula (11) can be solved with the method in [24]. If the solution satisfies *α*/*β* ≤ *r*_max⁡_, then it is the solution of formula (10); otherwise, the problem turns into solving formula (12).

It can be derived that *α*/*β* and the ellipse parameters satisfy the following equation:

(13) $$\begin{array}{c} \frac{\alpha }{\beta }=\sqrt{\frac{a+c+\sqrt{{\left(a-c\right)}^{2}+b^{2}}}{a+c-\sqrt{{\left(a-c\right)}^{2}+b^{2}}}}. \end{array}$$

After substituting the constraint 4*ac* − *b* ^2^ = 1 into equation (13), we can obtain

(14) $$\begin{array}{c} a+c=\frac{r_{\max}^{2}+1}{2r_{\max}}. \end{array}$$

Let *s* = (*r*_max⁡_^2^ + 1)/(2*r*_max⁡_), and substitute (14) into (8) and (12); the problem can be simplified to the following formula:

(15) argmin⁡a ∑iG(xi,yi)2subject  to 4(s−c)c−b2=1,

where *G*(*x*, *y*) is

(16) $$\begin{array}{c} G\left(x,y\right)=bxy+c\left(y^{2}-x^{2}\right)+dx+ey+f+sx^{2}. \end{array}$$

According to the Lagrange multiplier method, we introduce the following Lagrange function:

(17) $$\begin{array}{c} L\left(x,y\right)=\underset{i}{\sum }G{\left(x_{i},y_{i}\right)}^{2}+\lambda \left[4\left(s-c\right)c-b^{2}-1\right] \end{array}$$

and we can obtain the solution of formula (16) by solving equation set (18).

Equation set (18) can be simplified to a quartic function, the roots of which can be directly calculated. By substituting the roots into formula (15), we can obtain the solution of formula (12).

In summary, there are two steps for the proposed method: first, the method in [24] is adopted to solve the optimization problem in formula (11). If the fitted ellipse satisfies *α*/*β* ≤ *r*_max⁡_, then return the ellipse directly; otherwise, solve the optimization problem in formula (12) with the process described in (14) to (18). The fitting results with *r*_max⁡_ = +*∞*, *r*_max⁡_ = 4, and *r*_max⁡_ = 2 are shown in Figure 8(c). Consider

(18) $$\begin{array}{c} \frac{\partial L}{\partial b}=\underset{i}{\sum }2x_{i}y_{i}G\left(x_{i},y_{i}\right)-2b\lambda =0\\ \frac{\partial L}{\partial c}=\underset{i}{\sum }2\left(y_{i}^{2}-x_{i}^{2}\right)G\left(x_{i},y_{i}\right)+\lambda \left(4s-8c\right)=0\\ \frac{\partial L}{\partial d}=\underset{i}{\sum }2x_{i}G\left(x_{i},y_{i}\right)=0\\ \frac{\partial L}{\partial e}=\underset{i}{\sum }2y_{i}G\left(x_{i},y_{i}\right)=0\\ \frac{\partial L}{\partial f}=\underset{i}{\sum }G\left(x_{i},y_{i}\right)=0\\ \frac{\partial L}{\partial \lambda }=4\left(s-c\right)c-b^{2}-1=0. \end{array}$$

## 3. Experimental Results

To evaluate the performance of the proposed method, we test the method with a set of synthetic and real images. All of the experiments are performed on a Core I3 3.3 GHz PC with 2 GB DDR III SDRAM. The parameters of the method are selected as follows.

1. Parameters *d*_max⁡_ and *θ*_min⁡_ restrict the spatial relationship between the two line segments to be merged. A larger *d*_max⁡_ allows more line segment pairs to be considered, but the incorrect merging ratio and computational complexity increase seriously; a larger *θ*_min⁡_ prevents line segments with sharp intersection angles from being merged, but it increases the mismerging ratio. We set *d*_max⁡_ = 3 and *θ*_min⁡_ = 105° in the experiments.
2. Parameter *LC*_min⁡_ restricts the minimum number of line segments for a legal elliptical arc. A larger *LC*_min⁡_ effectively reduces the number of elliptical arcs, making the method more efficient at the price of a higher false negative ratio. We choose *LC*_min⁡_ = 2 in the experiments.
3. Parameters *a*_min⁡_, *a*_max⁡_, *r*_max⁡_, and *Th*_len_ restrict the properties of ellipses to be detected.

By setting these parameters appropriately, the false negative and false positive ratios can be significantly reduced. We set *a*_min⁡_ = 8, *a*_max⁡_ = 150, *r*_max⁡_ = 4, and *Th*_len_ = 1/3 in the experiment.

To better analyze the characteristics of the proposed method, we adopt some other successful methods as references.

1. The random Hough transform (RHT) [11]: the Hough transform is a widely used shape detector and has many variants for ellipse detection. Among these, the RHT is a representative method.
2. The Upwrite method (UPW) [17]: the UPW method is an edge-following method that is very efficient and has a good accuracy.
3. The methods in [19, 20]: these two methods are relatively new edge-following methods that have good performance both in accuracy and efficiency. We refer to the two methods as KIM and MAI, respectively.

### 3.1. Experiments on Synthetic Images

We design an ellipse-generating program to evaluate the accuracy of the proposed method. The program randomly generates *N* ellipses in a 300-by-300 black image. The center of each ellipse is arbitrarily located within the image. The semimajor axis of each ellipse is in the range of (10,150), the ratio of the semimajor axis and semiminor axis is not greater than 4, and the tangential angle is in the range of [0, *π*). Two sample images with *N* = 8 and *N* = 24 are shown in Figures 10(a)–10(g). Because all of the pixels that do not belong to an ellipse are outliers for the ellipse, the mean ratio of the outliers for all of the ellipses is (*N* − 1)/*N*. Therefore, the robustness to different ratios of outliers can be evaluated by using different values of *N*.

To measure the relationship of a detected ellipse *E*_*d*_ and a true ellipse *E*_*t*_, we adopt the “overlap error” in [9], as follows:

(19) $$\begin{array}{c} \text{Overlap}\;\text{Error}\left(E_{d},E_{t}\right)=1-\frac{\text{Area}\left(E_{t}\right)\cap \text{Area}\left(E_{d}\right)}{\text{Area}\left(E_{t}\right)\cup \text{Area}\left(E_{d}\right)}, \end{array}$$

where Area(*E*_*i*_) denotes the area of ellipse *E*_*i*_. A smaller overlap error indicates a higher detection accuracy. A detected ellipse *E*_*d*_ is considered to be correct if there is a true ellipse *E*_*t*_ that satisfies that the overlap error of the two ellipses is smaller than 0.05. We evaluate the performance of a method that has a recall ratio and precision ratio defined as follows:

(20) $$\begin{array}{c} \text{recall}=\frac{\text{number}\;\text{of}\;\text{correctly}\;\text{detected}\;\text{ellipses}}{\text{number}\;\text{of}\;\text{true}\;\text{ellipses}}\\ \text{precision}=\frac{\text{number}\;\text{of}\;\text{correctly}\;\text{detected}\;\text{ellipses}}{\text{number}\;\text{of}\;\text{detected}\;\text{ellipses}}. \end{array}$$

**Figure 9:**
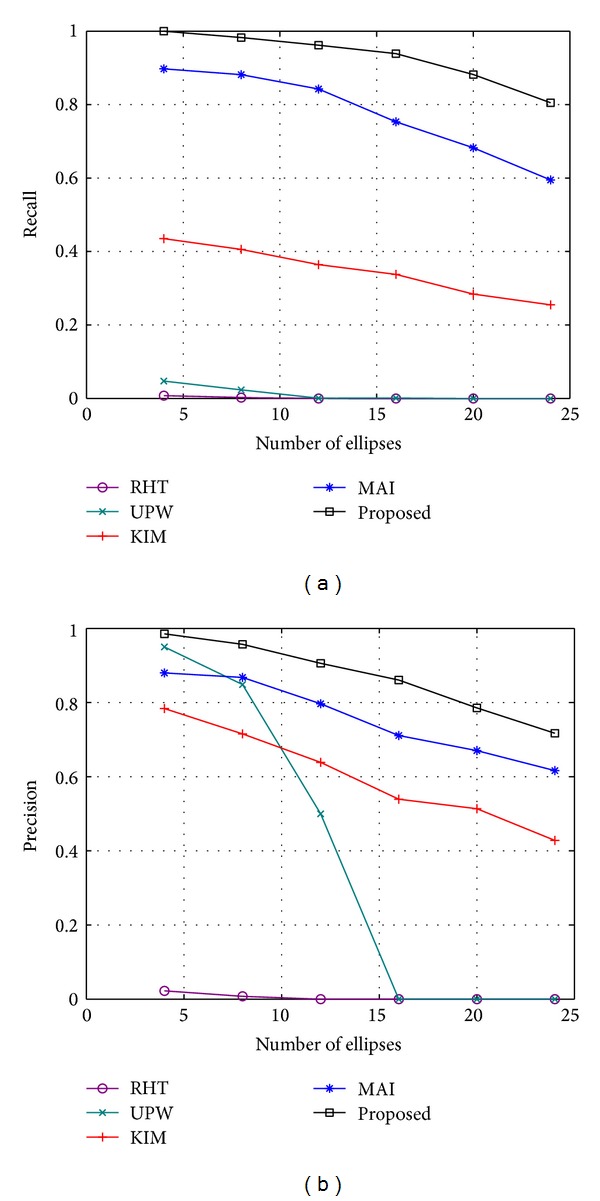
Recall and precision of each method for different number of ellipses. (a) Recall. (b) Precision.

**Figure 10:**
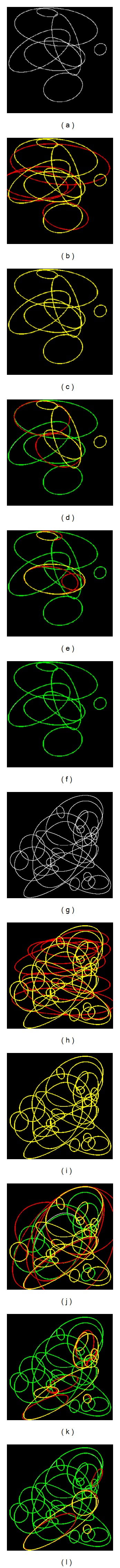
Two sets of detection results by each method. (a, g) are two images with *N* = 8 and *N* = 24, (b, h), (c, i), (d, j), (e, k), and (f, l) are the detection results by RHT, UPW, KIM, MAI, and the proposed method, respectively, where green ellipses are correct detections, yellow ellipses are false negatives, and red ellipses are false positives.

The recall and precision of each method for different values of *N* are shown in Figure 9. Each value in Figure 9 is the mean of the result of 100 randomly generated synthetic images (the same as all of the following experiments on synthetic images). It can be found that the recall and precision decrease for all of the methods as *N* increases, but the proposed method obtains the highest scores for recall and precision in almost all of the cases. For *N* = 8, the proposed method achieves high scores of 98.3% and 95.7% for the recall and precision, respectively. While the two scores are still 80.5% and 71.7% for *N* = 24, they are obviously better than the best reference method (MAI), with the two scores of 59.5% and 61.6%. This result indicates that the proposed method is more robust to the interference of outliers. Two sets of detection results from each method for *N* = 8 and *N* = 24 are shown in Figure 10.

**Figure 11:**
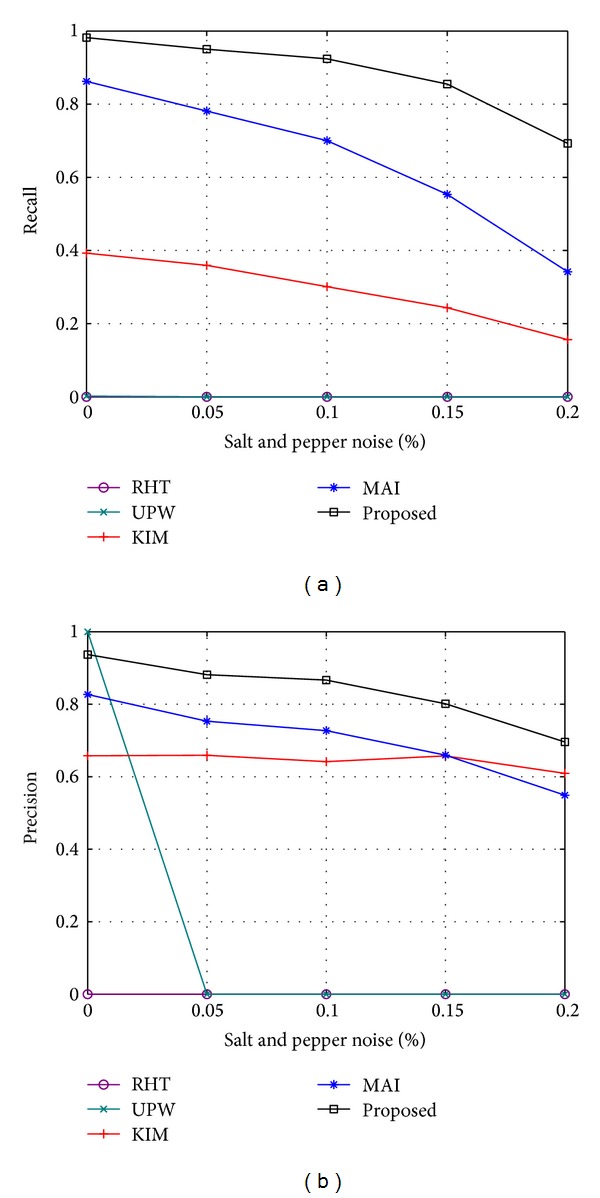
Recall and precision of each method for different ratios of salt and pepper noise. (a) Recall. (b) Precision.

**Figure 12:**
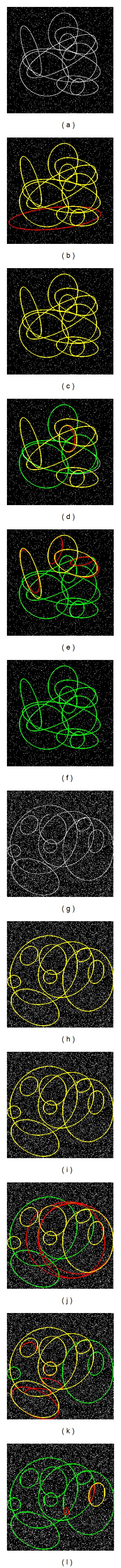
Two sets of detection results by each method. (a, g) are two images with 5% and 20% salt and pepper noise, (b, h), (c, i), (d, j), (e, k), and (f, l) are the detection results by RHT, UPW, KIM, MAI, and the proposed method, respectively, where the meaning of each color is the same as Figure 10.

To evaluate the robustness to noise of the proposed method, we add different ratios of salt and pepper noise on synthetic images. Salt and pepper noise can interrupt the elliptical curves or can generate fake branches, which is a great challenge for edge-following methods.  Figure 11 shows the recall and precision from each method for different ratios of salt and pepper noise, where *N* is fixed at 10. There, the proposed method obtains the highest recall and precision in almost all of the cases. Two sets of detection results from each method are shown in Figure 12.

**Figure 13:**
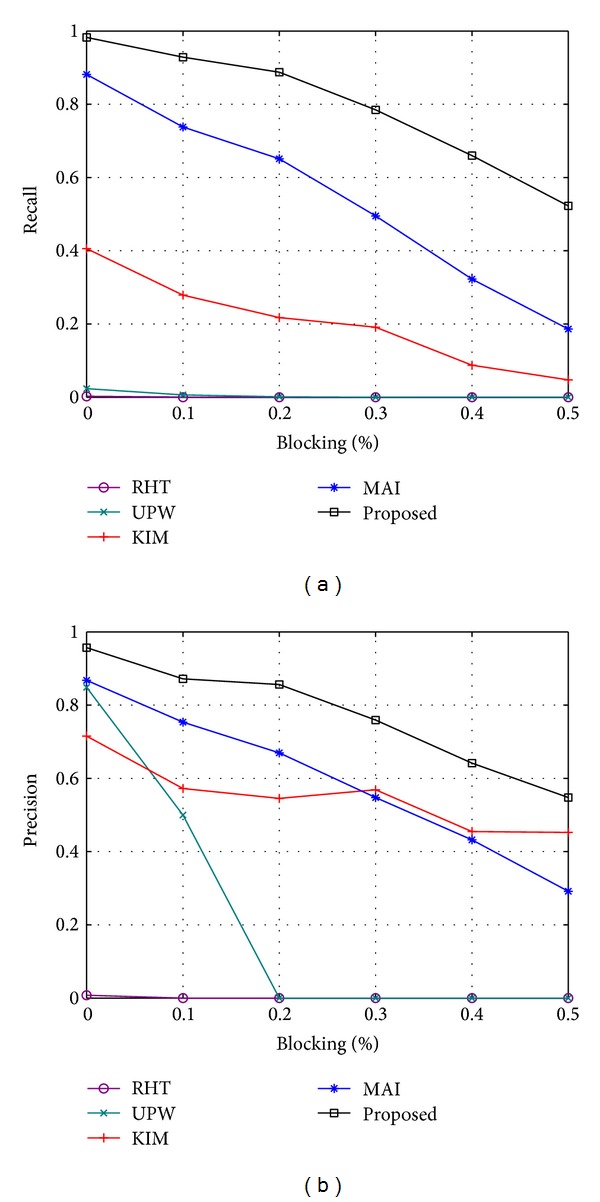
Recall and precision of each method for different ratios of occlusion (a) Recall. (b) Precision.

**Figure 14:**
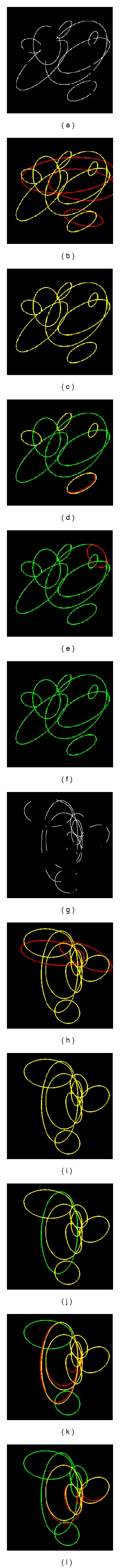
Two sets of detection results by each method. (a, g) are two images with 10% and 50% blocked, (b, h), (c, i), (d, j), (e, k), and (f, l) are the detection results by RHT, UPW, KIM, MAI, and the proposed method, respectively, where the meaning of each color is the same as Figure 10.

To evaluate the robustness to partial occlusion for the proposed method, we randomly generate a set of rectangles and remove all of the edge pixels in the region of the rectangles. The restriction is that at least 50% of the edge pixels are preserved for each ellipse; otherwise, the rectangles are generated again. We define the ratio *R*_*b*_ of occlusion as follows:

(21) $$\begin{array}{c} R_{b}=\frac{\text{Area}\left[\left(\cup_{i}\text{El}{\text{l}}_{i}\right)\cap \left(\cup_{j}\text{Rec}{\text{t}}_{i}\right)\right]}{\text{Area}\left(\cup_{i}\text{El}{\text{l}}_{i}\right)} \end{array}$$

which is the ratio of the area of all of the ellipses being blocked and the total area of the ellipses.  Figure 13 shows the recall and precision of each method for different occlusion ratios, where *N* is fixed at 8. Again, the proposed method obtains the highest recall and precision in almost all of the cases. Two sets of detection results from each method are shown in Figure 14.

**Figure 15:**
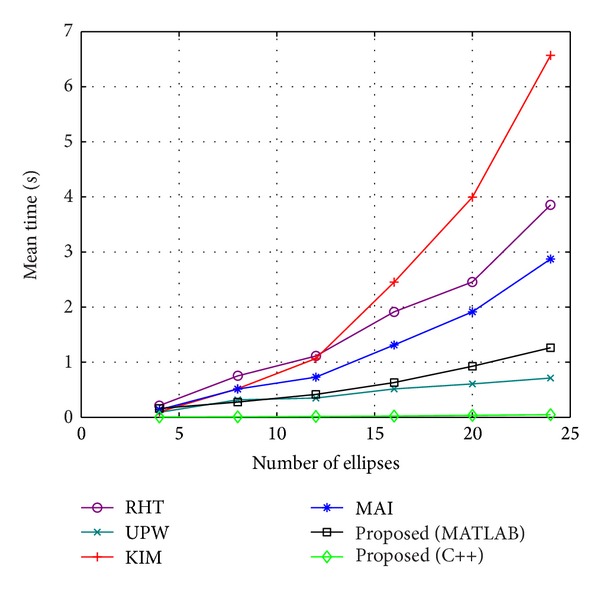
Time requirement of each method to process images with different number of ellipses.

Except for the detection accuracy, we also test the efficiency of the proposed method.  Figure 15 shows the time required for each method to process images with different values of *N*. The methods of RHT and UPW are implemented with the C programming language, the methods of KIM and MAI are implemented with MATLAB, and two versions of the proposed method are implemented with C++ and MATLAB.  Figure 15 shows that the time required by the proposed method is close to that of KIM and MAI in the case of *N* = 4. However, the time requirement of the proposed method increases much more slowly than KIM and MAI, as *N* increases. In the case of *N* = 24, the proposed method is approximately 2.3 times faster than MAI and 5.2 times faster than KIM. Although the time required by UPW is less than for the MATLAB version of the proposed method, the recall and precision of UPW are too low, as shown in Figure 9. In addition, the C++ version of the proposed method is approximately 30 times faster than the MATLAB version, which is also faster than the UPW method.

### 3.2. Real Images

**Figure 16:**
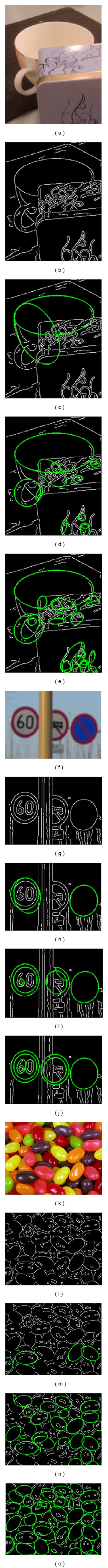
Detection results for real images by each method. (a, f, k) are original images, (b, g, l) are the extracted edge bitmaps, and (c, h, m), (d, i, n), and (e, j, o) are detection results by KIM, MAI, and the proposed method, respectively.

Because it is impossible for synthetic images to simulate all of the real situations, we test the proposed method with a set of real images. The detection results of KIM, MAI, and the proposed method for three real images are shown in Figure 16.

Figures 16(a)–16(e) show that all of the three methods detected the salient ellipse at the mouth of the cup. However, KIM failed to detect the ellipses at the handle of the cup, and MAI detected one of the ellipses, while the proposed method detected all of the three ellipses. There are many elliptical arcs on the box in Figure 16(a), most of which are detected by the proposed method, while much fewer ellipses are detected by KIM and MAI.

The detection results in Figures 16(f)–16(i) reflect the robustness to partial occlusion of the three methods. The sign on the right side is not blocked, and all of the three methods detected it correctly. The sign on the left side is slightly blocked, and only the result of KIM is not accurate. The sign in the middle is blocked seriously, and only the proposed method detected both external and internal ellipses. This result indicates that the proposed method is more robust to partial occlusion, which coincides with the results for synthetic images.

The image in Figure 16(k) is very complex; arcs interfere and block each other, and the arcs are not standard elliptical arcs. KIM detected only a few beans, and MAI detected more, while the proposed method detected most of the beans, which indicates that the proposed method has a higher recall for complex images.

The time requirement of the methods for the three images is listed in Table 1. It can be seen that the proposed method is much faster than KIM and MAI, and the C++ version of the proposed method is fast enough for real-time applications.

## 4. Conclusions

A fast and robust ellipse-detection method based on sorted merging is proposed in this paper. This method first links edge pixels into line segments; next, it gradually merges the line segments into elliptical arcs and candidate ellipses; then, it verifies the candidate ellipses to obtain the final results. Compared with other ellipse-detection methods, the proposed method has the following characteristics.

1. A sorted merging strategy is proposed for the merging process of the line segments and elliptical arcs. By merging the line segment/elliptical arc pairs in the descending order of the merging degrees, the merging accuracy is significantly increased.
2. Multiple properties of an ellipse are used to filter the line segment pairs and elliptical arc pairs, which makes the proposed method very efficient.
3. An ellipse-fitting method is proposed that restricts the maximum ratio of the semimajor axis and semiminor axis of the fitted ellipse. The merging accuracy is further improved with the proposed fitting method.

To evaluate the proposed method, we test it with many synthetic images and real images and compare the detection results with other methods. The experimental results indicate that the proposed method is robust to outliers, noise, and partial occlusion, and in most cases, the recall and precision of the proposed method are obviously higher than for other methods. In addition, the proposed method is more efficient than other methods, and the C++ version of the proposed method can be used in real-time applications.

However, the proposed method sometimes misses ellipses in images with very low quality. The reason is that the edge bitmap cannot be extracted accurately in such cases, which further influences the extraction accuracy of the line segments. We plan to improve the line segment extracting method to make it more robust to low-quality images.

## Figures and Tables

**Table 1:** Time requirement of each method to process the real images (s).

	KIM	MAI	Proposed (MATLAB)	Proposed (C++)
Figure 16(a)	0.916	1.686	0.408	0.047
Figure 16(f)	0.381	0.479	0.209	0.063
Figure 16(k)	5.027	4.434	2.243	0.109
